# Aberrantly hydroxymethylated differentially expressed genes and the associated protein pathways in osteoarthritis

**DOI:** 10.7717/peerj.6425

**Published:** 2019-02-25

**Authors:** Yang Fang, Pingping Wang, Lin Xia, Suwen Bai, Yonggang Shen, Qing Li, Yang Wang, Jinhang Zhu, Juan Du, Bing Shen

**Affiliations:** 1 School of Basic Medical Sciences, Anhui Medical University, Hefei, Anhui, China; 2 Nursing Faculty, Anhui Health College, Chizhou, Anhui, China; 3 Central Laboratory of Medical Research Center, Anhui Provincial Hospital, Hefei, Anhui, China

**Keywords:** DNA hydroxymethylation, Expression profile, Bioinformatics, Cartilage, Collagen

## Abstract

**Background:**

The elderly population is at risk of osteoarthritis (OA), a common, multifactorial, degenerative joint disease. Environmental, genetic, and epigenetic (such as DNA hydroxymethylation) factors may be involved in the etiology, development, and pathogenesis of OA. Here, comprehensive bioinformatic analyses were used to identify aberrantly hydroxymethylated differentially expressed genes and pathways in osteoarthritis to determine the underlying molecular mechanisms of osteoarthritis and susceptibility-related genes for osteoarthritis inheritance.

**Methods:**

Gene expression microarray data, mRNA expression profile data, and a whole genome 5hmC dataset were obtained from the Gene Expression Omnibus repository. Differentially expressed genes with abnormal hydroxymethylation were identified by MATCH function. Gene ontology and Kyoto Encyclopedia of Genes and Genomes (KEGG) pathway enrichment analyses of the genes differentially expressed in OA were performed using Metascape and the KOBAS online tool, respectively. The protein–protein interaction network was built using STRING and visualized in Cytoscape, and the modular analysis of the network was performed using the Molecular Complex Detection app.

**Results:**

In total, 104 hyperhydroxymethylated highly expressed genes and 14 hypohydroxymethylated genes with low expression were identified. Gene ontology analyses indicated that the biological functions of hyperhydroxymethylated highly expressed genes included skeletal system development, ossification, and bone development; KEGG pathway analysis showed enrichment in protein digestion and absorption, extracellular matrix–receptor interaction, and focal adhesion. The top 10 hub genes in the protein–protein interaction network were COL1A1, COL1A2, COL2A1, COL3A1, COL5A1, COL5A2, COL6A1, COL8A1, COL11A1, and COL24A1. All the aforementioned results are consistent with changes observed in OA.

**Conclusion:**

After comprehensive bioinformatics analysis, we found aberrantly hydroxymethylated differentially expressed genes and pathways in OA. The top 10 hub genes may be useful hydroxymethylation analysis biomarkers to provide more accurate OA diagnoses and target genes for treatment of OA.

## Introduction

Osteoarthritis (OA), a common degenerative joint disease, is associated with biochemical, metabolic, and morphological changes of the tissues, mainly in articular cartilage (Liao et al., 2015). Articular cartilage is composed and maintained primarily by chondrocytes and the extracellular matrix (ECM). Under normal conditions, chondrocytes are responsible for maintaining a balance between anabolic and catabolic factors while the ECM of the cartilage goes through continuous remodeling to maintain homeostasis (Ni et al., 2015). In OA, chondrocytes morphology and function and cartilage homeostasis changes causes overall matrix degradation (Blanco, Rego & Ruiz-Romero, 2011).

Epigenetics investigates the heritable changes in gene expression when the DNA sequence is unchanged (Bird, 2002). In most diseases, epigenetic alterations include, but is not limited to, DNA methylation, histone modification, etc. An oxidative product of 5-methylcytosine, 5-hydroxymethylcytosine (5hmC), is an intermediate in the active DNA demethylation pathway. Increasing evidence shows that 5hmC is not only an intermediate for DNA demethylation but also exists as an independent, stable epigenetic marker to influence gene expression (Bachman et al., 2014; Karlsson et al., 2010; Wu et al., 2011). In patients with OA, the global 5hmC levels was significantly increased compared to normal chondrocytes and associated with key OA genes expression (Taylor et al., 2015). Therefore, 5hmC may be importantly involved in the development of OA and potentially diagnostic and therapeutic targets (Taylor et al., 2014).

Microarray systems and high-throughput sequencing are efficient tools for examining gene expression and genetic or epigenetic variation and for identifying biomarkers in clinical studies (Soon, Hariharan & Snyder, 2013). However, many previous studies have used only one of these methods, making it difficult to verify the key genes and pathways involved in multiple cellular processes and biological functions. Overlapping several types of related data using bioinformatics analysis of databases may provide more reliable and accurate results (Liu et al., 2017a). Although numerous studies examining OA gene expression profiles and methylation profiles have used bioinformatics analyses to screen hundreds of differentially expressed genes (DEGs) that may be involved in the development of OA (Chou et al., 2013; Fisch et al., 2018), few of these studies have focused on abnormal hydroxymethylation (Taylor et al., 2015). In the present study, data from a gene expression microarray (GSE51588), mRNA high-throughput sequencing (GSE114007), and high-throughput genome-wide 5hmC analyses (GSE64393) were integrated and analyzed using several bioinformatics tools. Aberrantly hydroxymethylated DEGs and their associated protein pathways were identified in OA to construct a protein–protein interaction (PPI) network and to determine hub genes. The findings using such an integrated approach have the potential not only to identify aberrantly hydroxymethylated genes and their protein pathways in OA but also to elucidate the underlying molecular mechanisms that coordinate the occurrence of OA, potentially opening therapeutic avenues for the epigenetic regulation of OA.

## Material and Methods

### Microarray analysis and high-throughput sequencing

Gene expression profiling datasets (GSE51588, GSE114007) and a gene methylolation analysis dataset (GSE64393) were obtained from the Gene Expression Omnibus repository (https://www.ncbi.nlm.nih.gov/geo/) at the National Center for Biotechnology Information. In total, subchondral bone obtained from 40 OA patients and 10 healthy donors were examined using the GeneChip expression profiling dataset GSE51588 (platform: GPL13497, Agilent-026652 Whole Human Genome Microarray 4 × 44 K v2 [Probe Name version]), and cartilage tissues obtained from 20 OA patients and 18 healthy donors were examined using the high-throughput sequencing mRNA expression profile dataset GSE114007 (platform: Illumina HiSeq 2000 [*Homo sapiens* ]; Illumina NextSeq 500 [*Homo sapiens*]). The genome-wide 5hmC profile dataset GSE64393 (platform: GPL11154 Illumina HiSeq 2000 [*Homo sapiens*]) included four human articular chondrocytes with OA and four total genomic DNA 5hmc datasets for articular chondrocytes without OA. The information of all samples was shown in Table 1.

**Table 1:** The information of samples in GSE51588, GSE64393 and GSE114007 datasets.

Item	GSE51588 Dataset		GSE64393 Dataset		GSE114007 Dataset	
	Normal	Osteoarthritis	Normal	Osteoarthritis	Normal	Osteoarthritis
Gender	F: 6; M: 3	F: 22; M: 18	F: 1; M: 1	F: 1; M: 3	F: 13; M: 5	F:12 ; M: 8
Age	38.4 ± 4.01	69.675 ± 1.40	30.5 ± 2.02	68.25 ± 3.15	36.6 ± 3.17	66.2 ± 1.64
Tissue	Subchondral bone from lateral or medial tibial plateau		Chondrocytes		Knee articular cartilage	
Nucleotide	Total RNA	Total RNA	Total RNA	Total RNA	Genomic DNA	Genomic DNA

### Data processing

**Figure 1:**
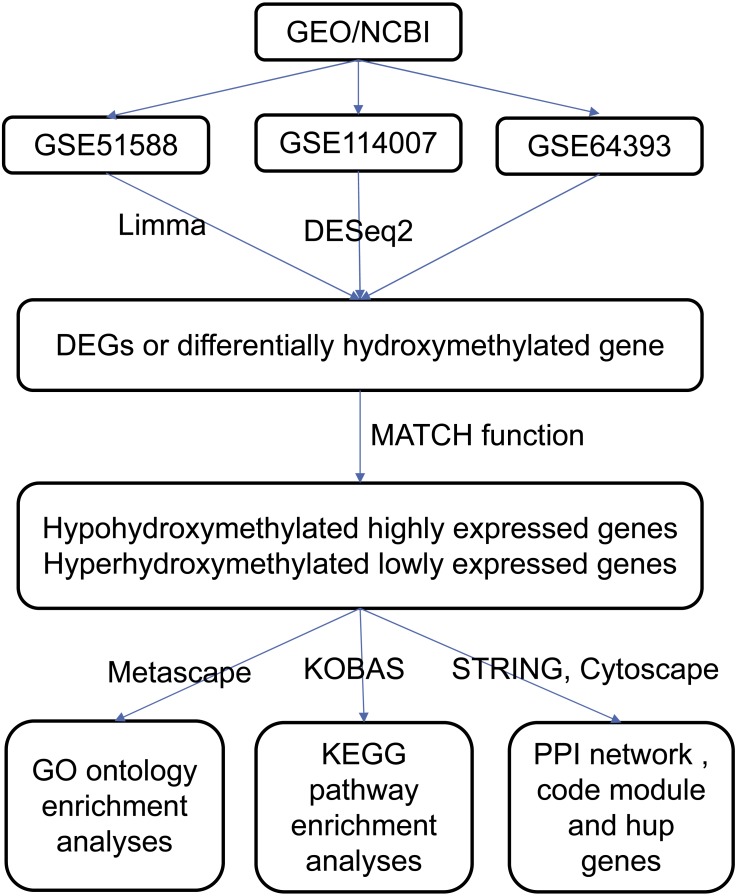
Flowchart of data analysis procedure.

The data were processed according to the flowchart (Fig. 1). The downloaded platforms and a series of matrix files were converted using R (v3.4.3) programming language and annotation software packages. The probe name was converted to the gene symbol of the corresponding gene and saved in a TXT file. Expression microarray datasets were analyzed using the Limma (v3.26.8) software package with default settings, and expression profile data was analyzed with the DESeq2 (v1.22.1) package with default settings (Varet et al., 2016). The whole genome 5hmC analysis result was obtained from the supplementary file of dataset of GSE64393. The selection criteria for DEGs and differentially hydroxymethylated genes were those with *P* values <0.05 and an absolute log2 (fold change) >1.

### Data integration

The MATCH function was used to find overlapping DEGs in the two gene expression profile datasets (GSE114007 and GSE51588), and those intersecting genes that were either upregulated or downregulated were identified (Fig. 1). In addition, the differentially hydroxymethylated genes were superimposed on the gene hydroxymethylation profile in the GSE64393 dataset. Finally, genes that were both hyperhydroxymethylated and upregulated were identified, and those that were both hypohydroxymethylated and downregulated were identified.

### Gene ontology and KEGG pathway enrichment analyses

Metascape, a web-based resource for gene annotation, visualization, and integration discovery (http://metascape.org) was used to perform functional and pathway enrichment analyses (Soonthornvacharin et al., 2017). Gene ontology (GO) analysis was performed using Metascape (Fig. 1). Kyoto Encyclopedia of Genes and Genomes (KEGG) pathway analysis of these aberrantly hydroxymethylated DEGs was performed using the KOBAS online analysis database (http://kobas.cbi.pku.edu.cn/) (Fig. 1). Values of *P* < 0.05 were considered statistically significant (Xie et al., 2011).

### PPI network construction and module analysis

PPI analysis may reveal the general organizational principles of functional cellular networks and provide new insights into protein function. The Search Tool for the Retrieval of Interacting Genes (STRING; http://string.embl.de/) provides information on the functional relationship between proteins (Fig. 1) (Von Mering et al., 2003). The PPI network associated with the respective aberrantly hydroxymethylated DEGs was constructed to predict the interaction of selected genes. Cytoscape (http://www.cytoscape.org/) is widely used to integrate biomolecular interaction networks with models to construct PPI networks of aberrantly hydroxymethylated DEGs (Fig. 1) (Lim et al., 2006). The Molecular Complex Detection (MCODE) app in Cytoscape was used to screen modules in the PPI network (Cao et al., 2018). Topology analysis was used to analyze the connectivity of the nodes in the PPI network to obtain a higher degree of key nodes (central proteins) (He & Zhang, 2006). The top 10 hub genes were selected for further analysis. Functional enrichment analysis of each module was performed using Metascape, with a significance threshold of *P* < 0.05.

## Results

### Identification of aberrantly hydroxymethylated DEGs in OA

**Figure 2:**
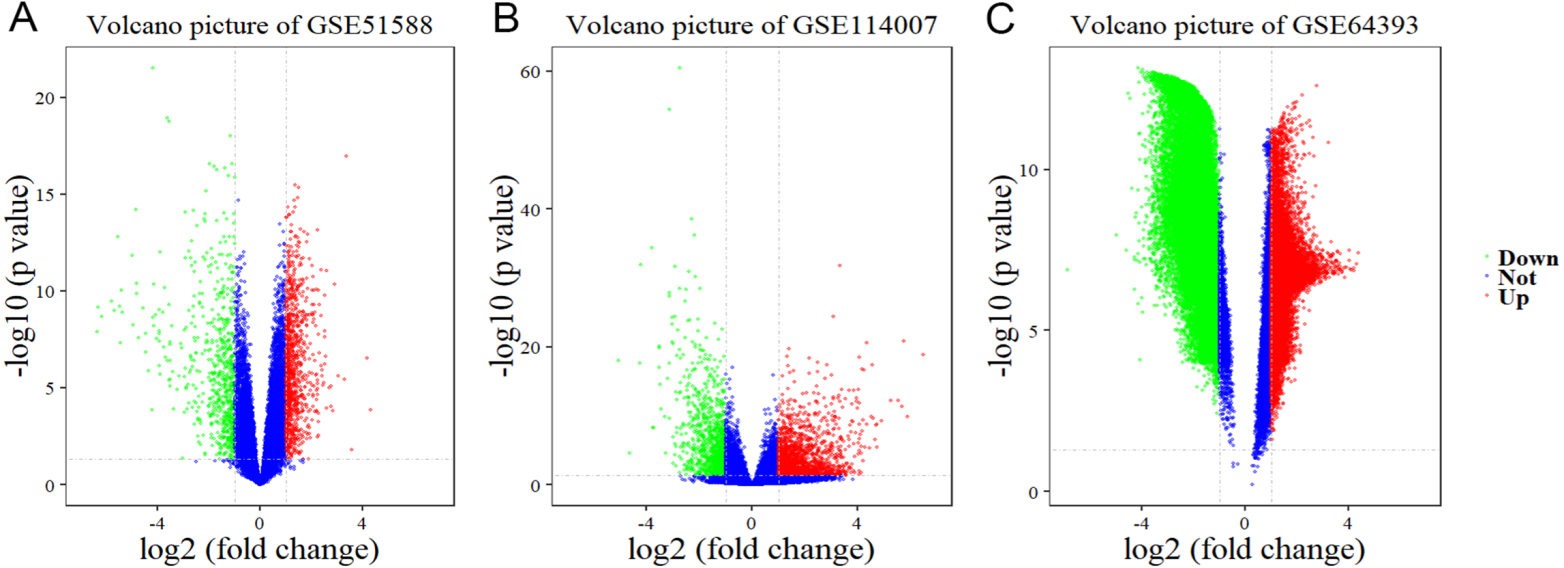
Differential gene expression and differential gene hydroxymethylation. (A) GSE51588 microarray dataset. (B) GSE114007 mRNA expression profile dataset. (C) GSE64393 high-throughput hydroxymethylation dataset. Red indicates upregulation, green indicates downregulation, and blue indicates no significant change in gene expression or hydroxymethylation based on the criteria of an absolute log2 (fold change) >1 and *P* < 0.05.

**Figure 3:**
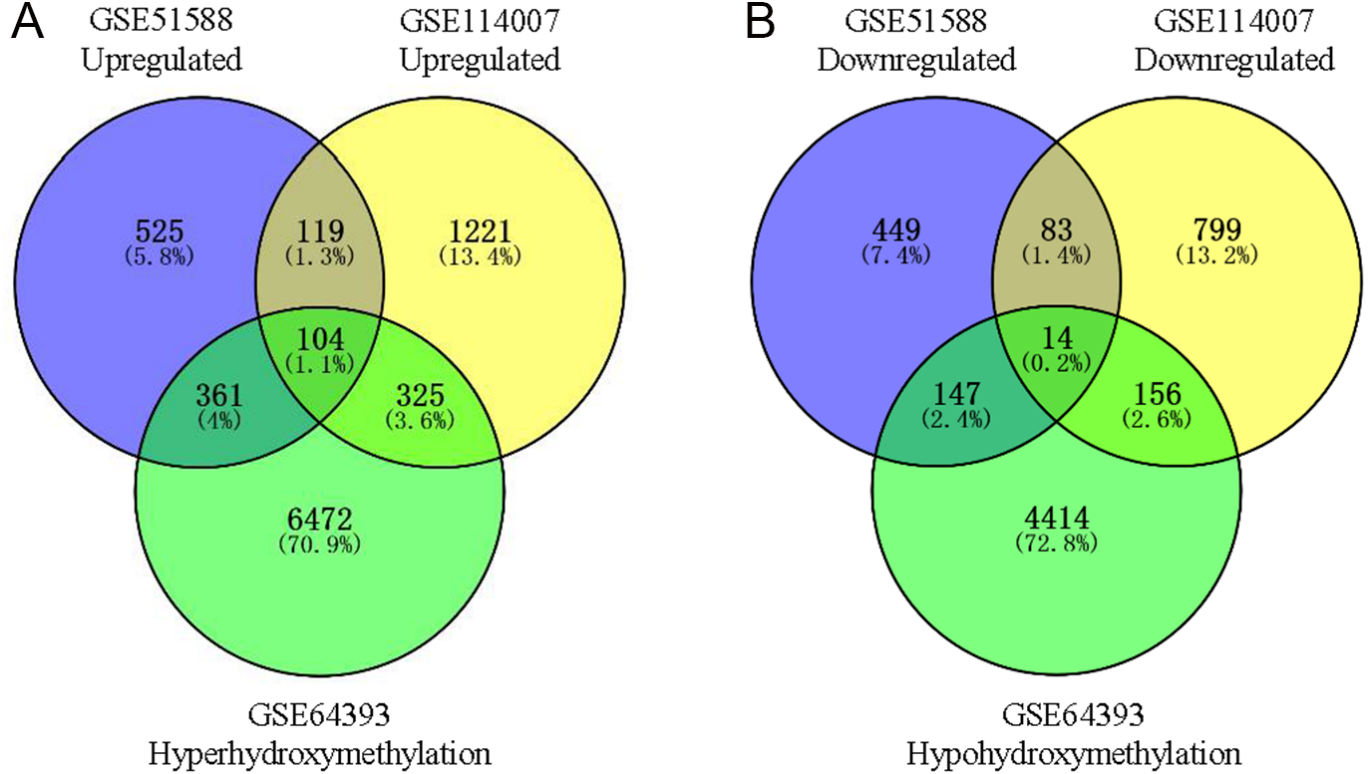
Identification of aberrantly hydroxymethylated differentially expressed genes in the gene expression datasets (GSE51588, GSE114007) and the gene hydroxymethylation dataset (GSE64393). (A) Hyperhydroxymethylation and upregulated genes; (B) hypohydroxymethylation and downregulated genes.

**Figure 4:**
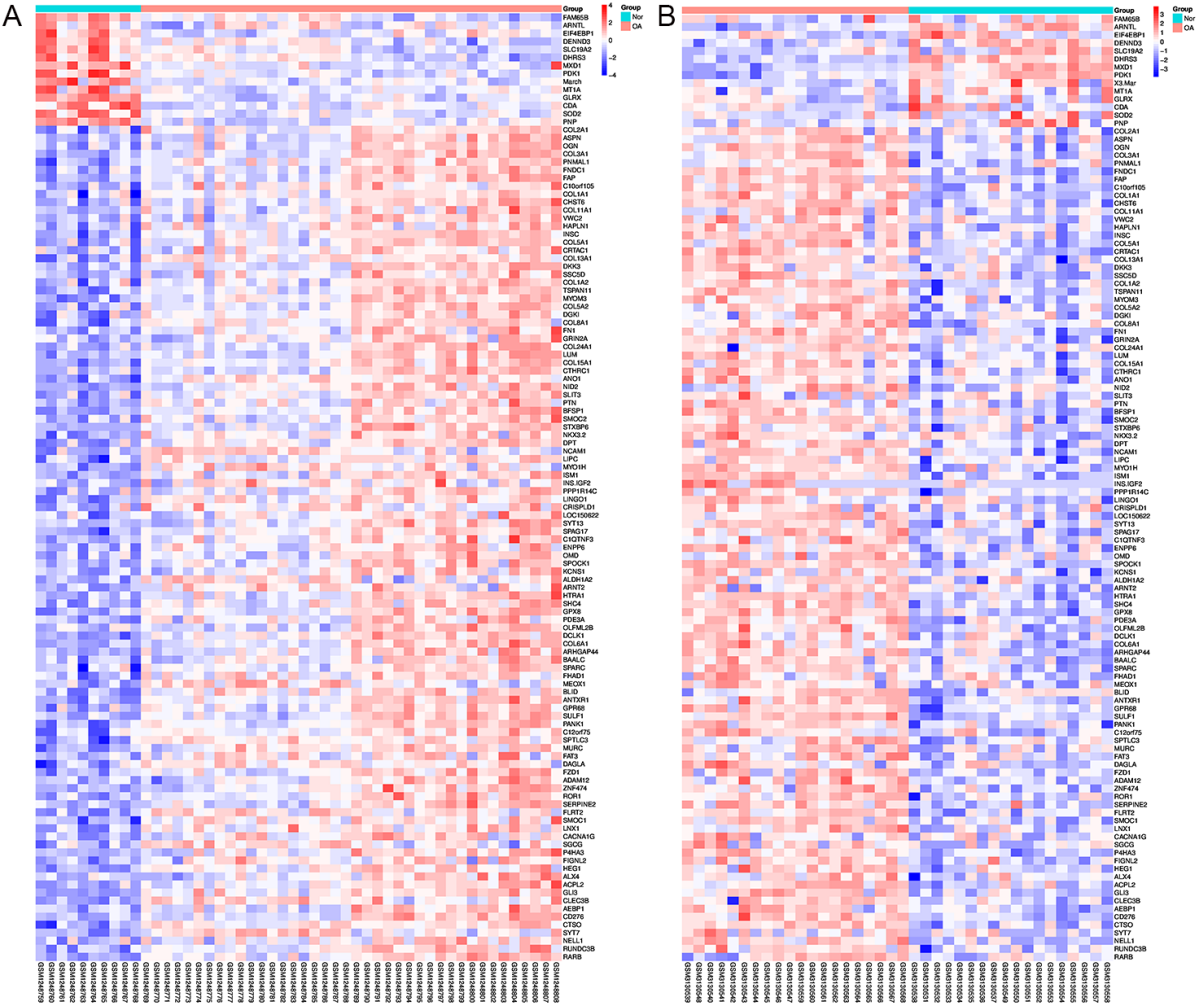
Heat maps of the aberrantly hydroxymethylated differentially expressed genes in the mRNA dataset. (A) GSE51588 dataset; (B) GSE114007 dataset. Red represents upregulation; blue, downregulation; N, normal; and OA, osteoarthritis.

Using Limma software to analyze the microarray GSE51588 system, we obtained 1,109 significantly upregulated DEGs and 693 significantly downregulated DEGs (Fig. 2A). Using the DESeq2 package to analyze the high-throughput sequencing mRNA expression profile dataset GSE114007, we obtained 1,769 significantly upregulated DEGs and 1,052 significantly downregulated DEGs (Fig. 2B). We then identified those DEGs contained in both gene expression profiles, finding 223 overlapping upregulated genes and 97 overlapping downregulated genes (Fig. 3). An analysis of the high-throughput hydroxymethylation dataset GSE64393 showed 7,262 hyperhydroxymethylated genes and 4,731 hypohydroxymethylated genes (Fig. 2C). We isolated the overlap of 7,262 hyperhydroxymethylated genes with the 223 upregulated genes. Then, a total of 104 hyperhydroxymethylated highly expressed genes were obtained. We overlapped the 4,731 hypohydroxymethylated genes and the 97 downregulated genes to identify 14 hypohydroxymethylated genes with low expression (Fig. 3). The heat maps for the expression of the aberrantly hydroxymethylated DEGs are shown in Fig. 4.

### GO functional enrichment analysis

**Figure 5:**
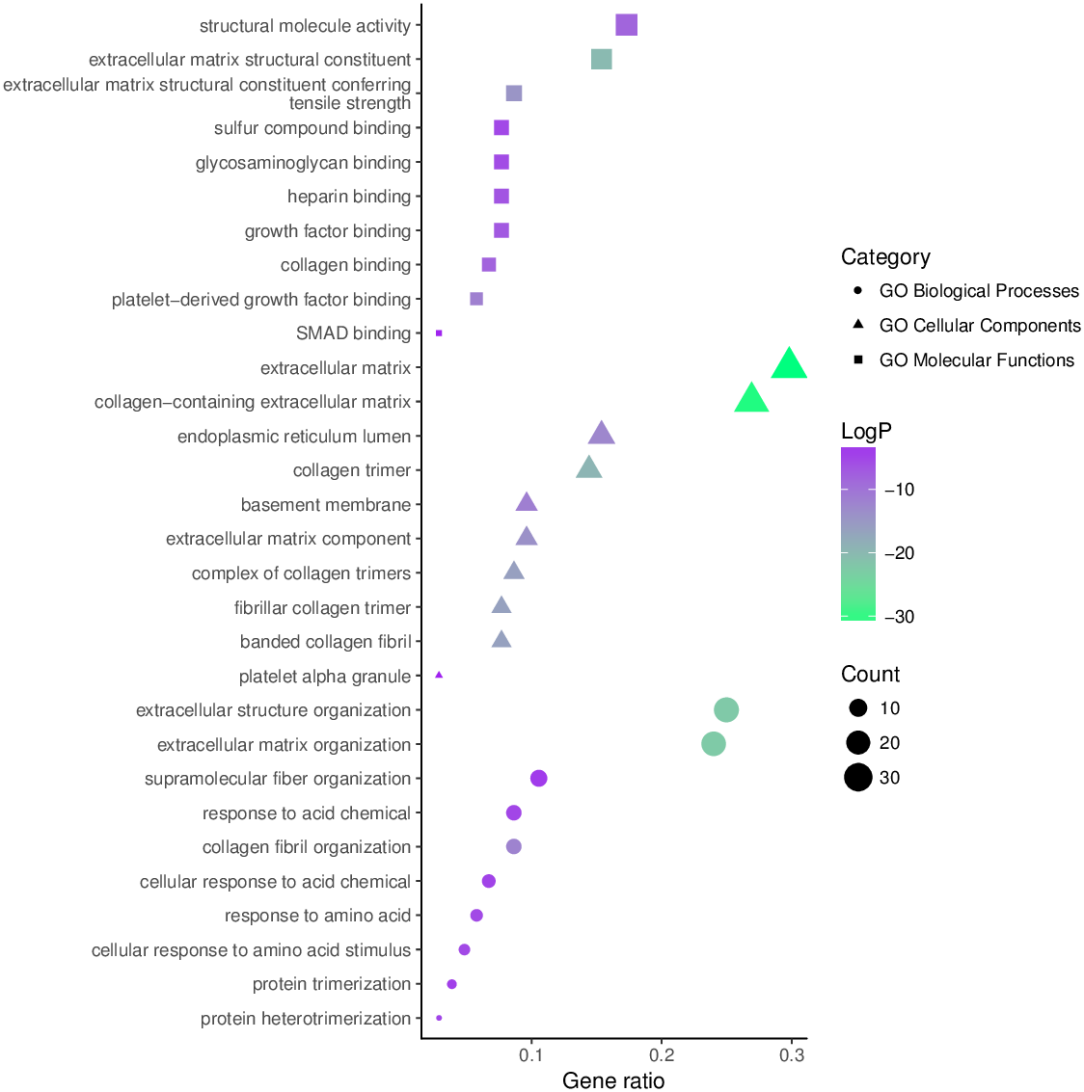
Enrichment of the gene ontology (GO) functional terms for the hyperhydroxymethylated highly expressed genes.

The top 10 significantly enriched GO terms, as determined using Metascape, are illustrated in Fig. 5. Hyperhydroxymethylated highly expressed genes were enriched in the biological processes of skeletal system development, ossification, bone development, blood vessel development, response to growth factor, and osteoblast differentiation. In the molecular function GO category, these genes showed enrichment in ECM structural constituent, collagen binding, calcium ion binding, ECM structural constituent conferring compression resistance, and ECM binding. The cell component GO category showed enrichment predominantly for the ECM, basement membrane, and synapse, indicating that hyperhydroxymethylated highly expressed genes may play key roles in the formation of the ECM and cartilage. For genes with hypohydroxymethylation and low expression, our Metascape findings indicated enrichments in the GO biological process category for cellular response to oxidative stress and nucleobase-containing small molecule metabolic process, and the GO molecular function category showed enrichment in oxidoreductase activity. These findings suggest that the upregulated genes may play a leading role in the etiology and development of OA.

### KEGG pathway analysis

**Figure 6:**
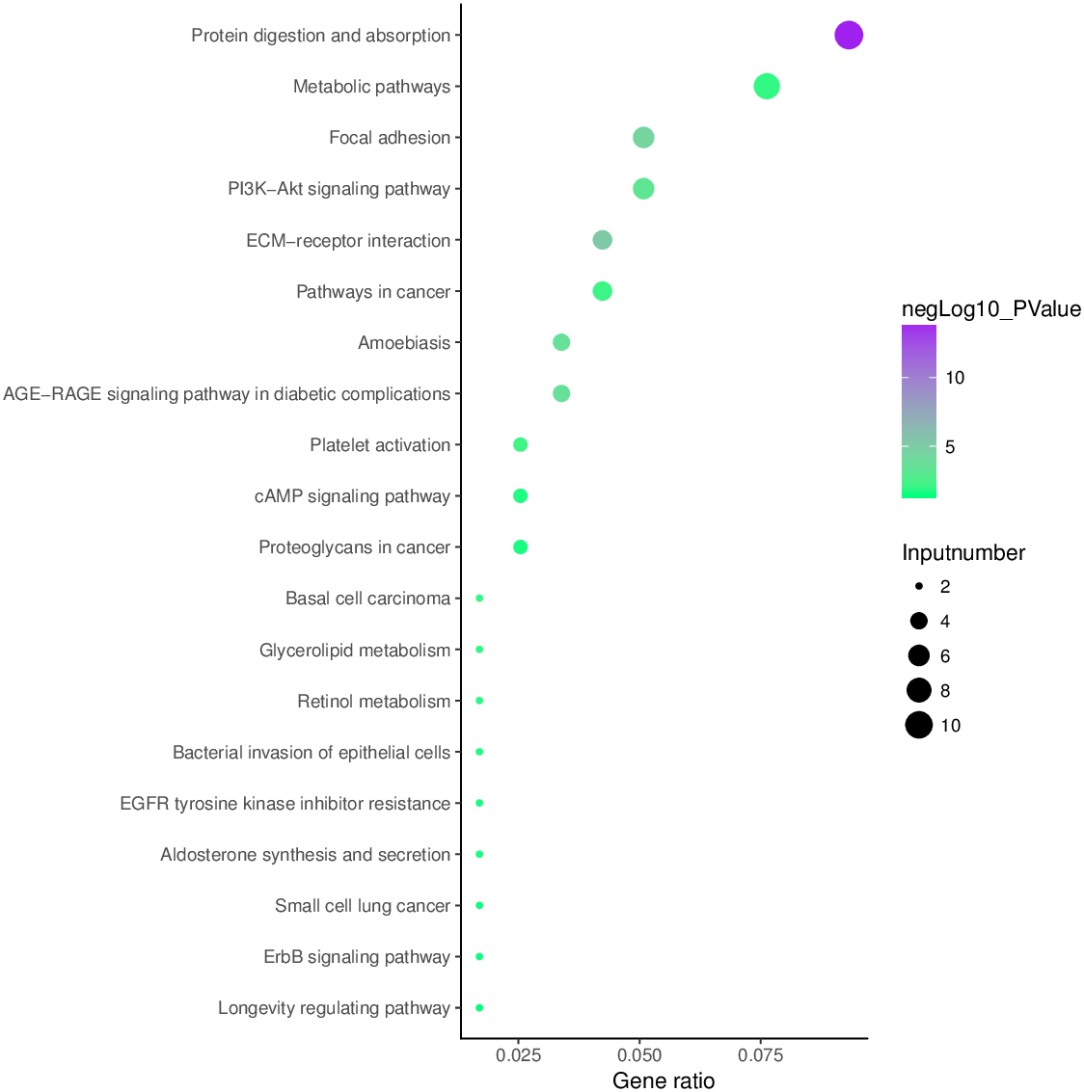
Enrichment of the aberrantly hydroxymethylated differentially expressed genes in Kyoto Encyclopedia of Genes and Genomes pathway analysis.

Analysis of aberrantly hydroxymethylated DEGs identified from the integration of gene microarray data in OA was conducted using the KOBAS online analysis tool. The top 10 KEGG pathway enrichment analysis results indicated that aberrantly hydroxymethylated DEGs were enriched in protein digestion and absorption, ECM–receptor interaction, focal adhesion, AGE–RAGE signaling pathway in diabetic complications, PI3K–Akt signaling pathway, platelet activation, pathways in cancer, metabolic pathways, basal cell carcinoma, and glycerolipid metabolism (Fig. 6).

### PPI network construction, module analysis, and hub gene selection

**Figure 7:**
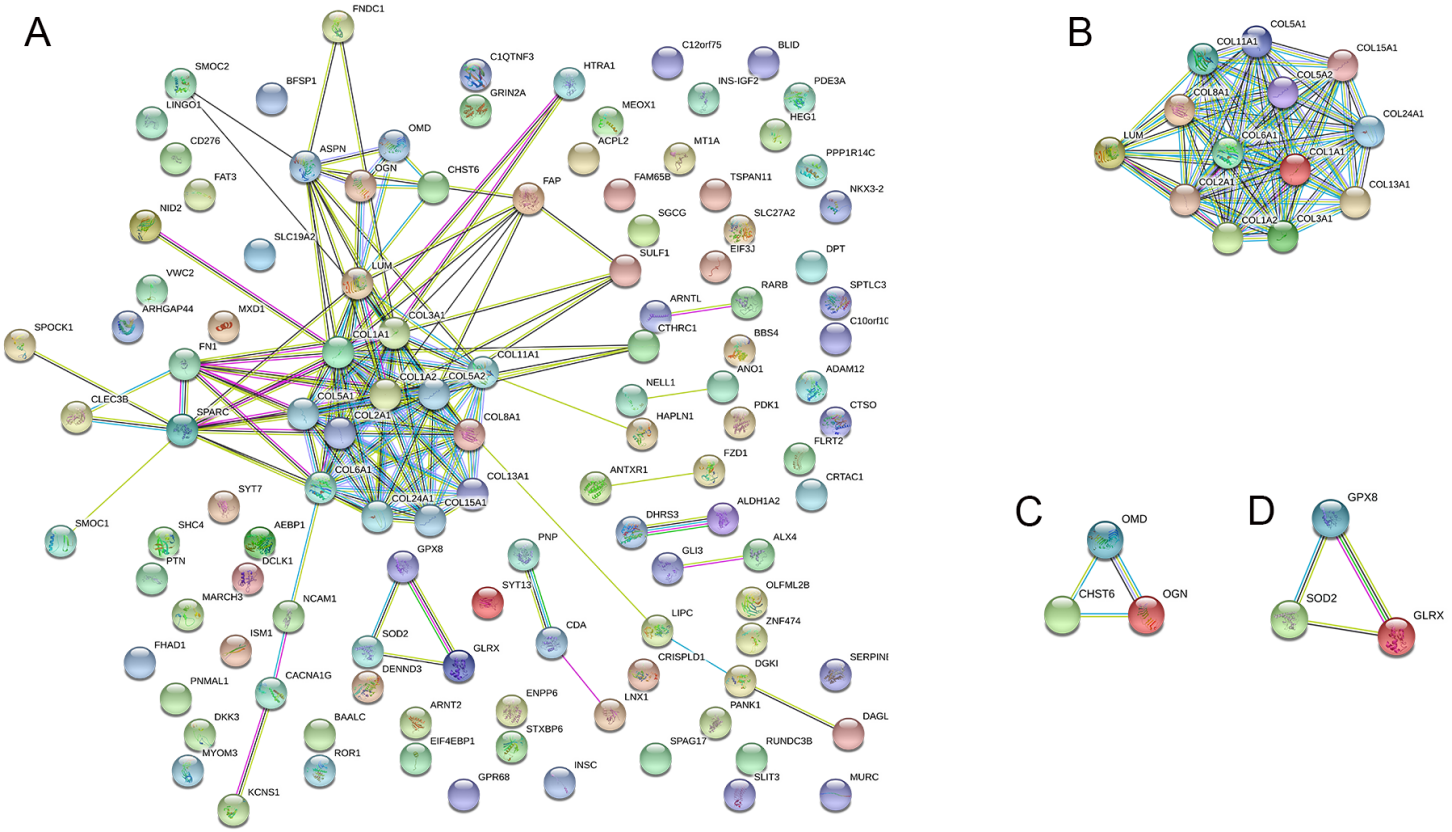
Protein–protein interaction network. Protein–protein interaction network (A) and the top three modules (B).

**Figure 8:**
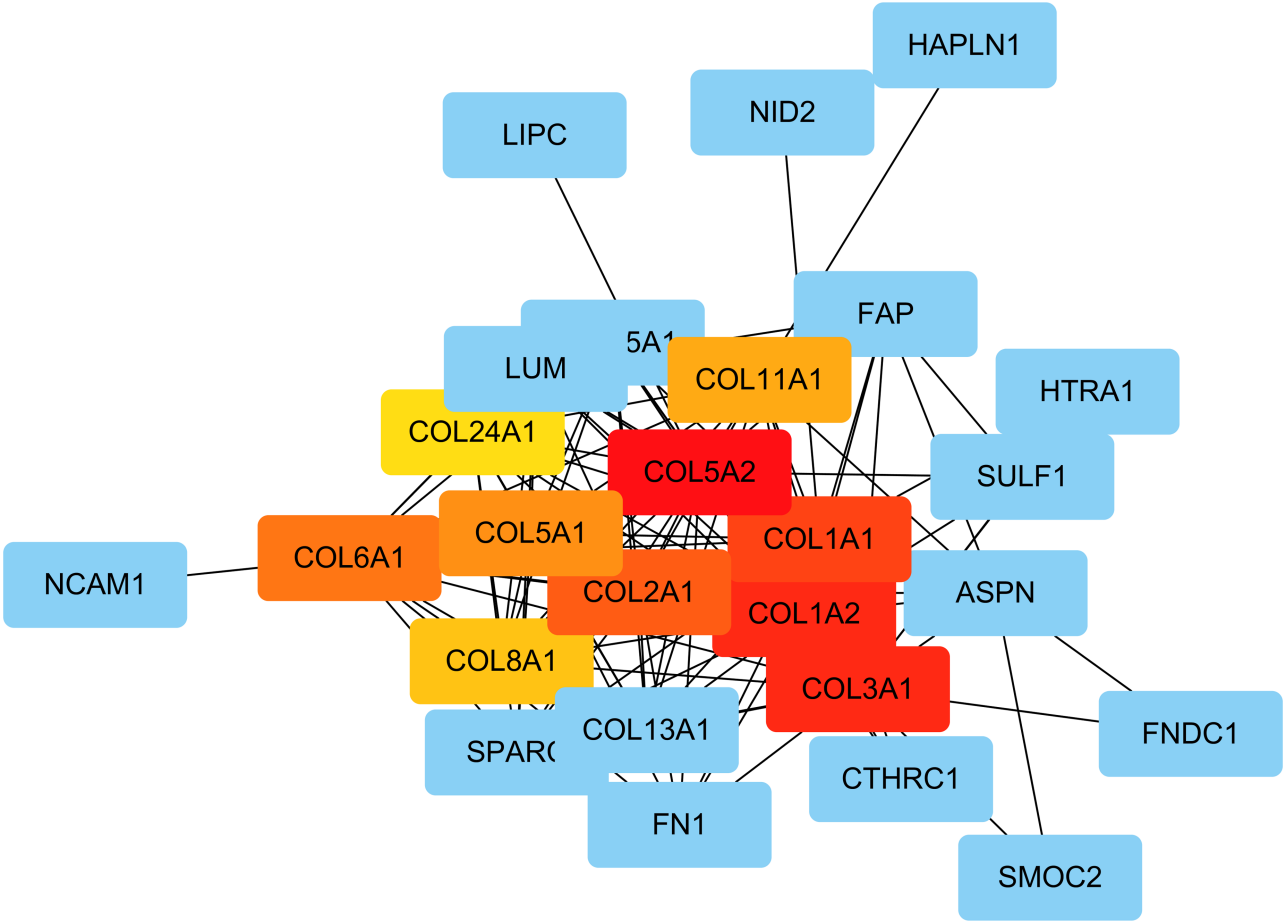
The top 10 hub genes. Blue indicates genes with low scores. Red indicates genes with high scores. Yellow and orange indicate genes with middle scores, increasing respectively.

The PPI network was created using the STRING website, and the function modules were analyzed using MCODE in Cytoscape. For the aberrantly hydroxymethylated DEGs, the PPI network is shown in Fig. 7A, and three modules are displayed in Fig. 7B. Important core modules showed collagen trimer, complex of collagen trimers, ECM structural constituent, glycosaminoglycan biosynthesis-keratan sulfate, and oxidoreductase activity. As showed in Fig. 8, the 10 genes with the most substantial interactions were COL1A1, COL1A2, COL2A1, COL3A1, COL5A1, COL5A2, COL6A1, COL8A1, COL11A1, and COL24A1, and all 10 core genes were hyperhydroxymethylated highly expressed genes.

## Discussion

Although OA affects approximately 40% of the elderly population, it is a growing public health problem worldwide because the genetic and molecular mechanisms underlying its occurrence and development remain unclear (Balakrishnan et al., 2014; Chapman & Valdes, 2012). Understanding the mechanisms underpinning OA development and progression will greatly contribute to the diagnosis, treatment, and prognosis of patients with this disease. Gene chip and high-throughput sequencing technologies can detect the expression levels of tens of millions of genes in humans and have been widely used to study the molecular mechanisms of various diseases, predict potential therapeutic targets, and find biomarkers (Dong et al., 2018; Liu et al., 2017a). Despite the numerous basic studies reporting on OA in recent years, the molecular mechanisms, early diagnosis, and epigenetic mechanisms of OA have not been elucidated because most of these studies have focused on the gene expression or pure methylation from a single cohort (Ren et al., 2018).

By contrast, the present bioinformatics study analyzed the mRNA expression profiles obtained from GeneChip data, transcriptome sequencing data, and a set of expression profile datasets from different groups of whole genome 5hmC data. Using MATCH analysis, we identified 118 aberrantly hydroxymethylated DEGs, including 104 hyperhydroxymethylated highly expression genes and 14 hypohydroxymethylated genes with low expression levels. The 118 aberrantly hydroxymethylated DEGs were categorized using the three GO functional annotations molecular functions, biological processes, and cellular components. DEG enrichment was determined using KEGG signal pathway analysis to construct a PPI of proteins that were encoded by these DEGs and to screen for the 10 most closely related genes.

Our results indicated that the aberrantly hydroxymethylated highly expressed DEGs in OA were involved mainly in the biological processes of skeletal system development, ossification, bone development, blood vessel development, response to growth factor, and osteoblast differentiation. These genes showed enrichment of molecular functions for ECM structural constituents, collagen binding, calcium ion binding, ECM structural constituent conferring compression resistance, and ECM binding. The cellular component enrichment for these genes was predominantly in the ECM, basement membrane, and synapse. These enriched functions and components are closely related to the growth of cartilage cells and the production of the cartilage that composes the ECM and are consistent with changes of the ECM and cartilage in patients with OA. In humans, the ECM consists mainly of proteins (especially collagen) and glycosaminoglycans (mainly proteoglycans) and is secreted by nearby cells. The ECM structural constituent, collagen binding, calcium ion binding, ECM structural constituent conferring compression resistance, ECM binding, ECM, basement membrane, and synapse are also closely related to the growth of cartilage cells as well as to the development and remodeling of cartilage. Our findings for these enrichments in OA suggests that collagen changes may play an important part in the development of OA. The hypohydroxymethylated genes with low expression levels were enriched in the cellular response to oxidative stress, nucleobase-containing small molecule metabolic processes, and oxidoreductase activity. The results of our KEGG signaling pathway analysis showed enrichments in protein digestion and absorption, ECM–receptor interaction. Thus, the results of our GO and KEGG enrichment analyses indicated that aberrantly hydroxymethylated DEGs are involved in numerous pathways associated with cartilage development and composition, consistent with the notion that these aberrantly hydroxymethylated DEGs play important roles in the occurrence and development of OA.

Our PPI network analyses of these aberrantly hydroxymethylated DEGs suggested that collagen trimer, complex of collagen trimers, ECM structural constituent, glycosaminoglycan biosynthesis-keratan sulfate, and oxidoreductase activity may be involved in OA development. These modules are closely related to the composition and homeostasis of cartilage and, again, demonstrate that collagen plays an important role in OA. We identified the top 10 hub genes in the PPI; the proteins encoded by these genes are the key nodes in the PPI network. We found that they were all highly methylolated, highly expressed genes encoding collagen and primarily involved in collagen trimer, collagen trimer complexes, and receptor tyrosine kinase signaling.

In the 10 core genes, increased mRNA expression levels of COL1A1, COL1A2, COL2A1, COL3A1, COL5A2 genes have been reported in OA (Bay-Jensen et al., 2018; Jin et al., 2009; Karlsson et al., 2010; Hermansson et al., 2004). Studies have shown that increased secretion of type I collagen encoded by COL1A1 and COL1A2 homotrimers in OA can induce stenosis and unorganized collagen fibers in subchondral bone (Clements et al., 2009; Couchourel et al., 2009; Jin et al., 2009; Steinberg et al., 2017). Type II collagen encoded by COL2A1, which is specific for cartilage tissue and is important for normal embryonic development of the bone, linear growth, and cartilage resistance to compression (Chen et al., 2016). Type III collagen encoded by COL3A1, together with type I collagen, provides a structural framework for the synovium surrounding the synovial joint (Hu et al., 2017; Reichert et al., 2018). Upregulation of type III in OA leads to thickening of the synovial membrane and has potential as a biomarker for OA (Bay-Jensen et al., 2018). Type V collagen encoded by COL5A1 and COL5A2, as a member of group I collagen, is present in tissues containing type I collagen, and appears to regulate the assembly of shaped fibers composed of type I and type V collagen (Colombi et al., 2017; Willard et al., 2018). Type XI collagen encoded by COL11A1 plays an important role in fiber formation by controlling the lateral growth of collagen II fibrils, since type XI collagen has previously been reported in the core of type II collagen and is believed to be useful in the stability of type II collagen (Liu et al., 2017b). The close interaction between type II and type XI collagen, which affects either of these mutations, may result in similar instability of cartilage tissue. Type XXIV collagen encoded by COL24A1, which may regulate the formation of type I collagen fibers during fetal development (Koch et al., 2003). The pathways associated with COL24A1 and the GO annotation linked with this gene include collagen chain trimerization, and ECM structural composition (Koch et al., 2003). These collagens have been reported to be closely related to cartilage composition and structural stability. Our results showed that the hydroxymethylation of these genes encoding collagens were significantly increased. Therefore, we believe that collagens may importantly involved in the development of OA.

## Conclusions

Using a combination of bioinformatics analysis of gene expression and of altered DNA hydroxymethylation, this study identified numerous aberrantly hydroxymethylated DEGs and their associated protein pathways in OA. These findings may help to identify the key genes and molecular mechanisms associated with OA initiation and development. The top 10 hub genes, COL1A1, COL1A2, COL2A1, COL3A1, COL5A1, COL5A2, COL6A1, COL8A1, COL11A1, and COL24A1, may be candidate biomarkers of hydroxymethylation abnormalities in OA that may be considered for more accurate diagnosis and treatment of OA. Compared with previous studies, the present study may provide more reliable and accurate screening results through our use of overlapping sets of related data. Although we have identified some candidate genes, further molecular experiments are still needed to discover other genes associated with OA.

##  Supplemental Information

10.7717/peerj.6425/supp-1 Supplemental Information 1 Differential gene expression and differential gene hydroxymethylation

10.7717/peerj.6425/supp-2 Supplemental Information 2 Enrichment of the gene ontology (GO) functional terms for the hyperhydroxymethylated highly expressed genes and hypohydroxymethylated lowly expressed genes

10.7717/peerj.6425/supp-3 Supplemental Information 3 Enrichment of the aberrantly hydroxymethylated differentially expressed genes in Kyoto Encyclopedia of Genes and Genomes pathway analysis

## Additional Information and Declarations

### Data Availability

The following information was supplied regarding data availability: Gene expression profiling datasets (GSE51588, GSE114007) and a gene methylation analysis data set (GSE64393) were obtained from the Gene Expression Omnibus repository (https://www.ncbi.nlm.nih.gov/geo/) at the National Center for Biotechnology Information.
