# Supplementary material for: Aberrantly hydroxymethylated differentially expressed genes and the associated protein pathways in osteoarthritis
Source: PeerJ. 2019 Feb 25;7:e6425. doi: 10.7717/peerj.6425 (PMC6394344; doi:10.7717/peerj.6425)
Supplement: Supplemental Information 2 [file peerj-07-6425-s002.zip › Supplemental Information 2/DOWN.zip!/Enrichment_heatmap/HeatmapSelectedGO.pdf]

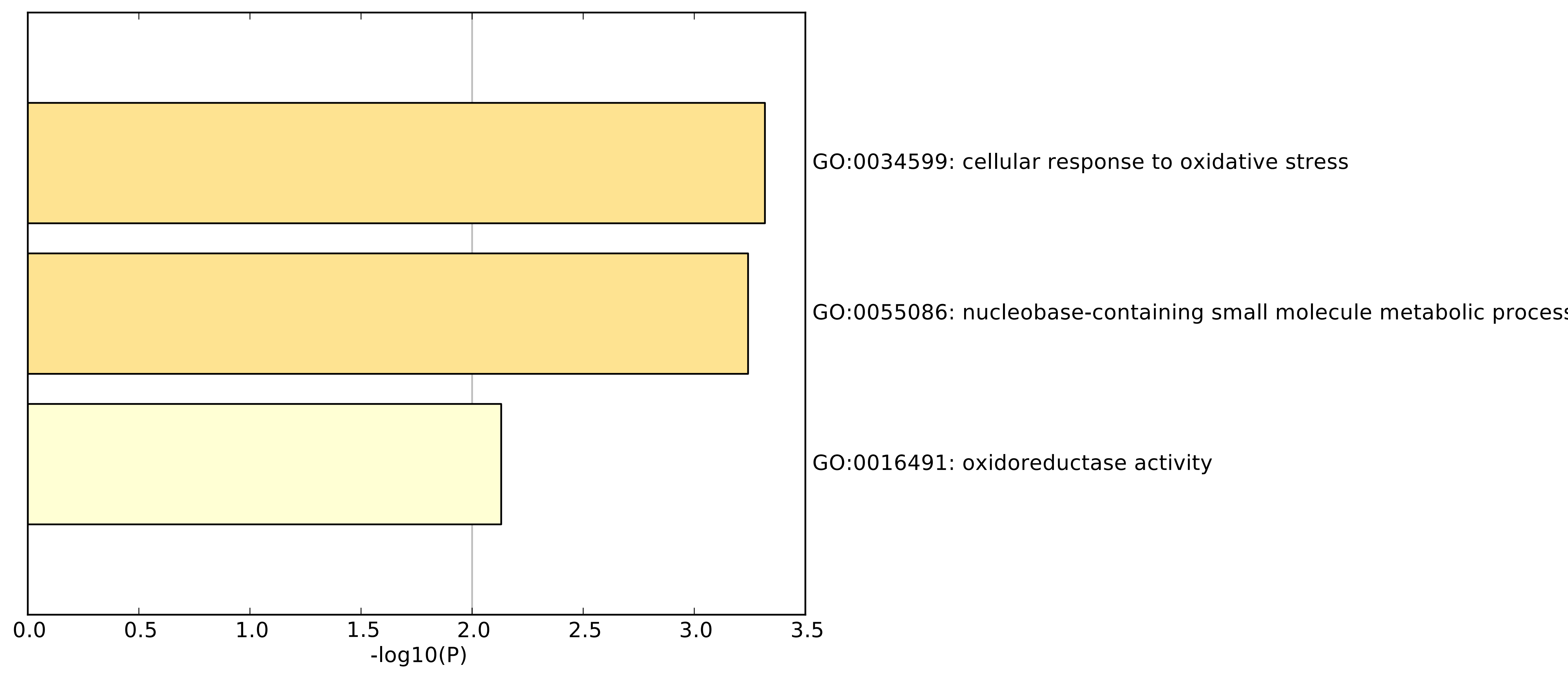

GO:0034599: cellular response to oxidative stress
GO:0055086: nucleobase-containing small molecule metabolic process
GO:0016491: oxidoreductase activity
0.0
0.5
1.0
1.5
2.0
2.5
3.0
3.5
-log10(P)
