# Supplementary material for: Aberrantly hydroxymethylated differentially expressed genes and the associated protein pathways in osteoarthritis
Source: PeerJ. 2019 Feb 25;7:e6425. doi: 10.7717/peerj.6425 (PMC6394344; doi:10.7717/peerj.6425)
Supplement: Supplemental Information 2 [file peerj-07-6425-s002.zip › Supplemental Information 2/UP.zip!/Enrichment_GO/ColorByCluster.pdf]

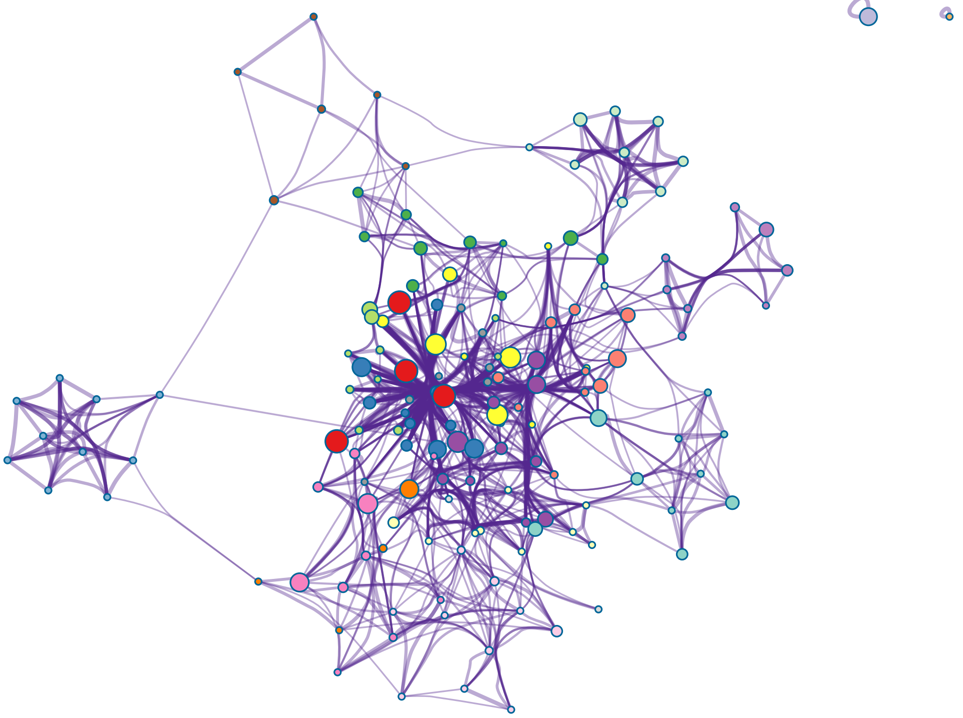

- extracellular matrix
- extracellular matrix structural constituent
- basement membrane
- skeletal system development
- ossification
- blood vessel development
- collagen binding
- response to growth factor
- endodermal cell differentiation
- heart morphogenesis
- bone development
- calcium ion binding
- head development
- extracellular matrix structural constituent conferring c[illegible]
- extracellular matrix binding
- wound healing
- osteoblast differentiation
- collagen metabolic process
- synapse part
- cell morphogenesis involved in differentiation

created by
http://metascape.org
