# Supplementary material for: Aberrantly hydroxymethylated differentially expressed genes and the associated protein pathways in osteoarthritis
Source: PeerJ. 2019 Feb 25;7:e6425. doi: 10.7717/peerj.6425 (PMC6394344; doi:10.7717/peerj.6425)
Supplement: Supplemental Information 2 [file peerj-07-6425-s002.zip › Supplemental Information 2/UP.zip!/Enrichment_GO/ColorByPValue.pdf]

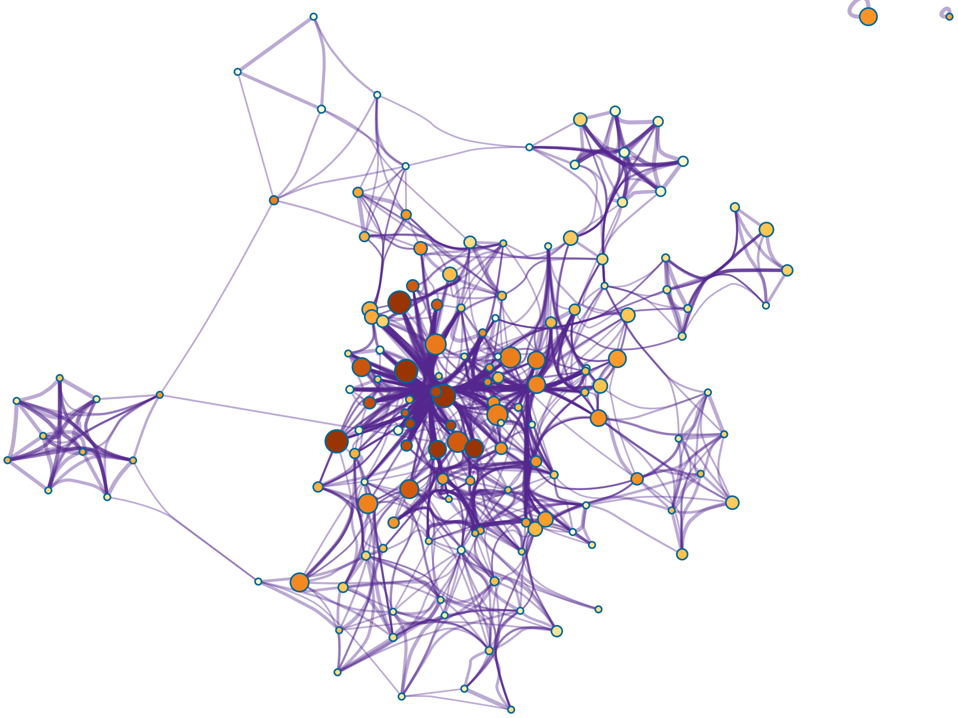

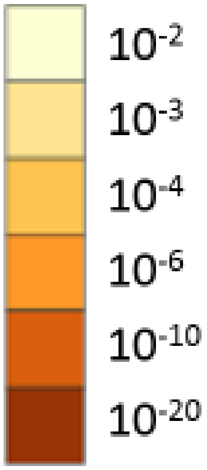

created by
http://metascape.org
