# Supplementary material for: Aberrantly hydroxymethylated differentially expressed genes and the associated protein pathways in osteoarthritis
Source: PeerJ. 2019 Feb 25;7:e6425. doi: 10.7717/peerj.6425 (PMC6394344; doi:10.7717/peerj.6425)
Supplement: Supplemental Information 2 [file peerj-07-6425-s002.zip › Supplemental Information 2/UP.zip!/Enrichment_heatmap/HeatmapSelectedGO.pdf]

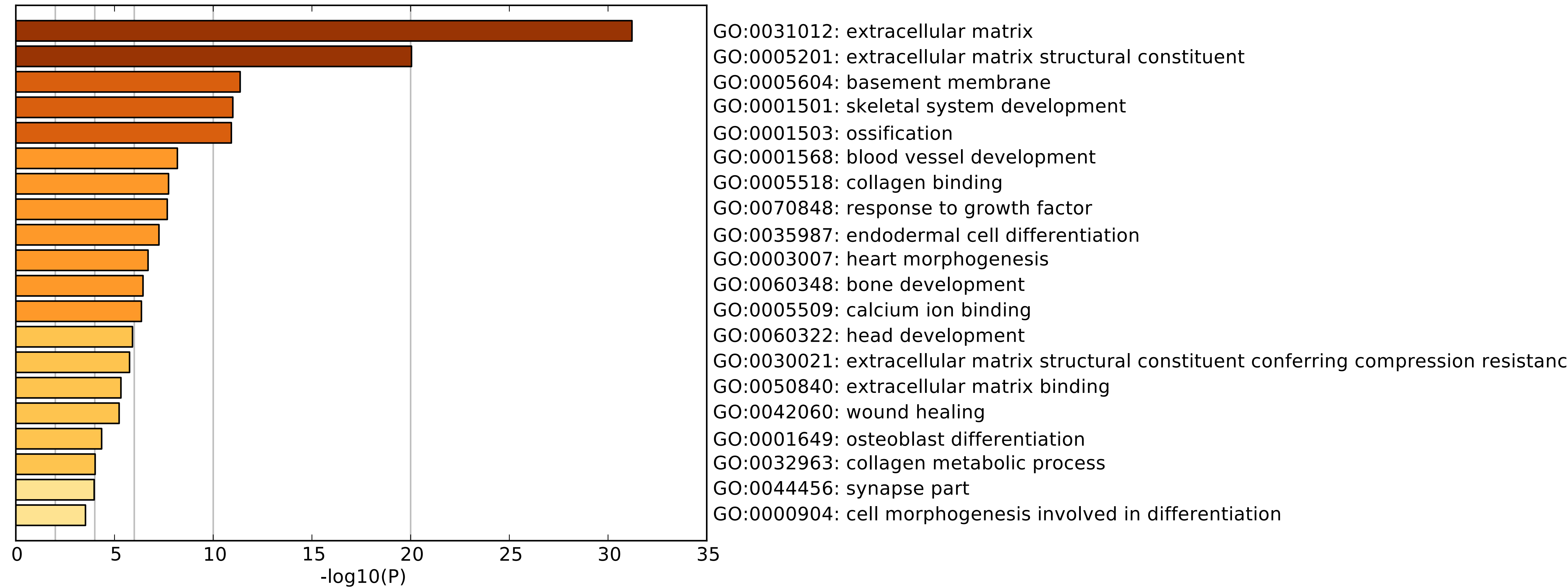

GO:0031012: extracellular matrix
GO:0005201: extracellular matrix structural constituent
GO:0005604: basement membrane
GO:0001501: skeletal system development
GO:0001503: ossification
GO:0001568: blood vessel development
GO:0005518: collagen binding
GO:0070848: response to growth factor
GO:0035987: endodermal cell differentiation
GO:0003007: heart morphogenesis
GO:0060348: bone development
GO:0005509: calcium ion binding
GO:0060322: head development
GO:0030021: extracellular matrix structural constituent conferring compression resistanc
GO:0050840: extracellular matrix binding
GO:0042060: wound healing
GO:0001649: osteoblast differentiation
GO:0032963: collagen metabolic process
GO:0044456: synapse part
GO:0000904: cell morphogenesis involved in differentiation
0
5
10
15
20
25
30
35
-log10(P)
