# Supplementary material for: Aberrantly hydroxymethylated differentially expressed genes and the associated protein pathways in osteoarthritis
Source: PeerJ. 2019 Feb 25;7:e6425. doi: 10.7717/peerj.6425 (PMC6394344; doi:10.7717/peerj.6425)
Supplement: Supplemental Information 2 [file peerj-07-6425-s002.zip › Supplemental Information 2/UP.zip!/AnalysisReport.html]

## Metascape Gene List Analysis Report

metascape.org^1^

### Enrichment Summary

Figure 1. Heatmap of enriched terms across input gene lists, colored by p-values.

### Gene Lists

User-provided gene identifiers are first converted into their H. sapiens Entrez gene IDs using the latest database (updated on 2018-01-01). If multiple identifiers correspond to the same Entrez gene ID, they will be considered as a single Entrez gene ID in downstream analyses. The gene lists are summarized in Table 1.

Table 1. Statistics of input gene lists.

| Name | Total | Unique |
| --- | --- | --- |
| Input ID | 104 | 104 |

### Pathway and Process Enrichment Analysis

For each given gene list, pathway and process enrichment analysis was carried out with the following ontology sources: GO Biological Processes, GO Cellular Components and GO Molecular Functions. All genes in the genome were used as the enrichment background. Terms with p-value < 0.05, minimum count 3, and enrichment factor > 1.5 (enrichment factor is the ratio between observed count and the count expected by chance) are collected and grouped into clusters based on their membership similarities. More specifically, p-values are calculated based on accumulative hypergeometric distribution^2^, q-values are calculated using the Banjamini-Hochberg procedure to account for multiple testing^3^. Kappa scores^4^ were used as the similarity metric when performing hierachical clustering on the enriched terms and then sub-trees with similarity > 0.3 are considered a cluster. The most statistically significant term within a cluster is chosen as the one representing the cluster.

Table 2. Top 20 clusters with their representative enriched terms (one per cluster). "Count" is the number of genes in the user-provided lists with membership in the given ontology term. "%" is the percentage of total user-provided genes that are found in the given ontology term (only input genes with at least one ontology term annotation are included in the calculation). "Log10(P)" is the p-value in log base 10. "Log10(q)" is the multi-test adjusted p-value in log base 10.

| GO | Category | Description | Count | % | Log10(P) | Log10(q) |
| --- | --- | --- | --- | --- | --- | --- |
| GO:0031012 | GO Cellular Components | extracellular matrix | 31 | 29.8076923077 | -31.2130767689 | -26.8690186965 |
| GO:0005201 | GO Molecular Functions | extracellular matrix structural constituent | 16 | 15.3846153846 | -20.044114346 | -16.3990262779 |
| GO:0005604 | GO Cellular Components | basement membrane | 10 | 9.61538461538 | -11.3643239056 | -8.19635709223 |
| GO:0001501 | GO Biological Processes | skeletal system development | 18 | 17.3076923077 | -10.993068883 | -7.85313079318 |
| GO:0001503 | GO Biological Processes | ossification | 16 | 15.3846153846 | -10.9180406928 | -7.80443154171 |
| GO:0001568 | GO Biological Processes | blood vessel development | 18 | 17.3076923077 | -8.18444152461 | -5.09565595726 |
| GO:0005518 | GO Molecular Functions | collagen binding | 7 | 6.73076923077 | -7.73717676043 | -4.75484652399 |
| GO:0070848 | GO Biological Processes | response to growth factor | 17 | 16.3461538462 | -7.67187695829 | -4.70803012755 |
| GO:0035987 | GO Biological Processes | endodermal cell differentiation | 6 | 5.76923076923 | -7.25108296658 | -4.32199824209 |
| GO:0003007 | GO Biological Processes | heart morphogenesis | 10 | 9.61538461538 | -6.69876878882 | -3.83183197109 |
| GO:0060348 | GO Biological Processes | bone development | 9 | 8.65384615385 | -6.44348851806 | -3.65573294638 |
| GO:0005509 | GO Molecular Functions | calcium ion binding | 15 | 14.4230769231 | -6.36308984889 | -3.5872335005 |
| GO:0060322 | GO Biological Processes | head development | 15 | 14.4230769231 | -5.90987962875 | -3.18907084669 |
| GO:0030021 | GO Molecular Functions | extracellular matrix structural constituent conferring compression resistance | 4 | 3.84615384615 | -5.76142920052 | -3.07058364185 |
| GO:0050840 | GO Molecular Functions | extracellular matrix binding | 5 | 4.80769230769 | -5.32479144311 | -2.66036270701 |
| GO:0042060 | GO Biological Processes | wound healing | 12 | 11.5384615385 | -5.23348543114 | -2.57962343871 |
| GO:0001649 | GO Biological Processes | osteoblast differentiation | 7 | 6.73076923077 | -4.34746380623 | -1.88285997956 |
| GO:0032963 | GO Biological Processes | collagen metabolic process | 5 | 4.80769230769 | -4.01782545956 | -1.59804667317 |
| GO:0044456 | GO Cellular Components | synapse part | 12 | 11.5384615385 | -3.96877136031 | -1.55413221357 |
| GO:0000904 | GO Biological Processes | cell morphogenesis involved in differentiation | 11 | 10.5769230769 | -3.52821651195 | -1.22555112466 |

To further capture the relationship among terms, a subset of enriched terms were selected and rendered as a network plot, where terms with similarity > 0.3 are connected by edges. Currently we select the terms with the best p-values from each of the 20 clusters, with the constraint that there are no more than 15 terms per cluster and no more than 250 terms in total. The network is visualized with Cytoscape^5^, where each node represented an enriched term and colored by its cluster ID (Figure 2.a) and then by p-value (Figure 2.b). These networks can be visualized interactively in Cytoscape with the .cys files (contained in the Zip package, which also contains publication-quality pdf version.) or within browser by clicking on the web icon. For clarity, term labels are only shown for one term per cluster, so it is recommended to use Cytoscape or browser to visualize the network in order to inspect all node labels. One can also export the network into pdf format within Cytoscape and then edit labels with Adobe Illustrator for publication purposes. To switch off all labels, delete the "Label" mapping under the "Style" tab within Cytoscape and then export network view.

Figure 2. Network of enriched terms: (a) colored by cluster ID, nodes share the same cluster are typically close to each other; (b) colored by p-value, terms containing more genes tend to have a more significant p-value.

|  |  |
| --- | --- |
|  |  |
|  |  |

### Protein-protein Interaction Enrichment Analysis

For each given gene list, protein-protein interaction enrichment analysis was carried out with the following databases: BioGrid^6^, InWeb\_IM^7^, OmniPath^8^. The resultant network contains the subset of proteins that form physical interactions with at least another list member. If the network contains 3 to 500 proteins, Molecular Complex Detection (MCODE) algorithm^9^ was further applied to identify densely connected network components. The MCODE networks identified for individual gene lists were pooled and shown in Figure 3.

Pathway and process enrichment analysis was applied to each MCODE components independently and the three best-scoring (by p-value) terms were retained as the functional description of the corresponding components, shown in a table underneath each network plot within Figure 3.

Figure 3. Protein-protein interaction network and MCODE components identified in the gene lists.

| GO | Description | Log10(P) |
| --- | --- | --- |
| GO:0062023 | collagen-containing extracellular matrix | -28.7 |
| GO:0031012 | extracellular matrix | -26.3 |
| GO:0098644 | complex of collagen trimers | -22.6 |

| Color | MCODE | GO | Description | Log10(P) |
| --- | --- | --- | --- | --- |
|  | MCODE\_1 | GO:0005788 | endoplasmic reticulum lumen | -5.7 |

### Reference

1. Tripathi S. et al., Meta- and Orthogonal Integration of Influenza "OMICs" Data Defines a Role for UBR4 in Virus Budding. Cell Host & Microbe (2015) 18:723-735.
2. Zar, J.H. Biostatistical Analysis 1999 4th edn., NJ Prentice Hall, pp. 523
3. Hochberg Y., Benjamini Y. More powerful procedures for multiple significance testing. Statistics in Medicine (1990) 9:811-818.
4. Cohen, J. A coefficient of agreement for nominal scales. Educ. Psychol. Meas. (1960) 20:27-46.
5. Shannon P. et al., Cytoscape: a software environment for integrated models of biomolecular interaction networks. Genome Res (2003) 11:2498-2504.
6. Stark C. et al. BioGRID: a general repository for interaction datasets. Nucleic Acids Res. (2006) 34:D535-539.
7. Li T. et al. A scored human protein-protein interaction network to catalyze genomic interpretation. Nat. Methods. (2017) 14:61-64.
8. Turei D. et al. A scored human protein-protein interaction network to catalyze genomic interpretation. Nat. Methods. (2016) 13:966-967.
9. Bader, G.D. et al. An automated method for finding molecular complexes in large protein interaction networks. BMC bioinformatics (2003) 4:2.
